# Obsessive-Compulsive Symptoms as a Manifestation of Homocystinuria

**DOI:** 10.1155/2021/5523453

**Published:** 2021-03-20

**Authors:** Vera Froes, Hugo Afonso, Zita Gameiro

**Affiliations:** ^1^Centro Hospitalar Barreiro Montijo, Barreiro, Portugal; ^2^Centro Hospitalar Tondela Viseu, Viseu, Portugal

## Abstract

Homocystinuria is a rare autosomal recessive metabolic disorder due to a defect in the cystathionine *β*-synthase (CBS) that leads to high homocysteine plasma levels. Psychiatric symptoms secondary to homocystinuria have been described in the literature; however, there is a lack of information about obsessive-compulsive symptoms correlated to this disorder. We describe the case of a 39 years old man, diagnosed with homocystinuria in childhood, with no previous psychiatric history that presented obsessive-compulsive disorder (OCD) like symptoms, as a manifestation of homocystinuria. This case underlines the importance for a psychiatrist to explore medical nonpsychiatric history, especially when presentation is abrupt, atypical, or in treatment-resistant cases.

## 1. Introduction

Homocystinuria (HCU) is a rare autosomal recessive metabolic disorder due to a defect in the cystathionine *β*-synthase (CBS) that leads to high homocysteine (Hcy) plasma levels (normal reference range is 5–15 mmol/L). Clinically, HCU results in a multisystem disorder with ocular, skeletal, cardiovascular, and central nervous system defects, though other organs can also be affected. Typical features, particularly in untreated patients, include early-onset severe myopia, ectopia lentis, osteoporosis, a tall thin marfanoid habitus, scoliosis, thromboembolic events in early adulthood, and variably decreased intelligence quotient (IQ) [1].

With respect to the central nervous system, stroke, mental retardation, and seizures have been described extensively. Psychiatric impairment has been documented [2–7]. In a retrospective study [2], 16 of the 25 patients in the sample (64%) reported psychiatric symptoms, including a high prevalence of both anxiety (32%) and depression (32%). Deficit–hyperactivity disorder (ADHD), oppositional defiant disorder (ODD), mood swings, hallucinations, and suicidal thoughts have also been reported. [2].

Less frequent, Abbott et al. found a 5% prevalence of OCD in a retrospective study with 63 patients with homocystinuria [3]. To our knowledge, there are no more recent studies reporting obsessive-compulsive symptoms related to HCU.

We report a case of a man who developed obsessive-compulsive symptoms induced by homocystinuria.

## 2. Case Presentation

A 39-year-old male, single, with no prior psychiatric history was admitted to the Emergency Department with behaviour changes and increased anxiety. In the last two months, he was restless, anxious, reporting multiple preoccupations “afraid of harming someone,” “afraid of losing control,” and “need to prove that everything is fine.” He also had severe insomnia and tiredness. Two weeks before, his worries increased in intensity and become intrusive, unwanted, and repetitive and he reported he was “afraid of forgetting important things,” saying that he had to write things down, as well as having difficulty of speaking because he was afraid to do a mistake or say something he did not want to, which led to him felling “blocked.” He showed a repetitive speech, centred about his work where he had verification behaviour, always confirming if he had done something wrong. That culminated with a feeling that he was losing control. He asked his parents to hide the knives because he was afraid of hurting someone.

The patient had a diagnosis of homocystinuria at the age of 16, with several complications like renal infarction, deep vein thrombosis, and retinal detachment. Although he had a history of failing two years in primary school, he managed to achieve the ninth grade of regular school education. He does not fulfil diagnostic criteria of intellectual disability because social, interpersonal, or practical domains of his life were not significantly compromised, as can be seen, namely, by a stable job in maintenance, social relationships, and no practical difficulties in daily living tasks.

He was medicated with warfarin, and no specific diet was followed. He had an aunt and a cousin with type 1 bipolar disorder. Psychiatric examination revealed an anxious mood, a speech centred in preoccupations about his work and people around him and prominent obsessive doubts like “Did I did it well?”, “Did I hurt someone?” or “Am I violent?” Vital signs were stable and the physical signs including neurological examination were unremarkable. Routine laboratory tests (including toxicology screening) and brain computed tomography (CT) scan were normal except for high levels of homocysteine (821 *μ*mol/l). Brain magnetic resonance imaging (MRI) showed low T2 signal, microvascular ischemia in white matter of the right frontal lobe.

First, he was given haloperidol 2 mg tid because of agitation. Then, he started a low protein diet and a treatment with cyanocobalamin 0.2 mg bid, pyridoxine 200 mg bid, thiamine 100 mg bid, and folic acid 5 mg bid. After two weeks of treatment, the symptoms were practically absent. At the time he was discharged, he still had some thoughts and worries about his work and verification behaviour leading to him confirming if he had done anything wrong, but he was able to return to work.

Two weeks after discharge, he attended an appointment and he was asymptomatic. Haloperidol was progressively reduced until stopped in about two weeks. A month after inpatient discharge, his homocysteine levels were lower (362.9 *μ*mol/l), and he remained asymptomatic. The treatment was changed to pyridoxine 300 mg tid that lead to undetectable levels in a month (<15 *μ*mol/l).

He was followed during a year and maintained on a low protein diet and pyridoxine 300 mg tid. After this period, he was discharged from psychiatry consultation.

## 3. Discussion

In the literature, there are some case reports that document psychiatric manifestations of homocystinuria, especially affective symptoms [4], psychotic symptoms [5], and impulsive behaviour [6]. In some cases, those symptoms were the initial presentation [7].

Although there has been hypothesised an association to obsessive-compulsive symptoms, to our knowledge, only three cases reports were described, by Abbott et al. in 1987 [3]. In all of them, patients were below thirties and had low IQ. As such, obsessive-compulsive symptoms as a manifestation of HCU seem rare.

Our patient did have some learning disabilities. Despite this fact, no formal evaluation was performed because other domains of his life were not impaired. He has a stable job in maintenance, social relationships, and no practical difficulties in daily living tasks.

As Almuqbil described in his study, all except one of the patients with homocystinuria with cognitive deficits experienced psychiatric symptoms. However, the presence of psychiatric symptoms was not a simple manifestation of low IQ, as those with higher IQ also experienced symptoms at a higher rate than the general population. [2]

Obsessive-compulsive disorder (OCD) has been described among mentally retarded individuals [8]. In these patients with intellectual disabilities, first encountered symptoms are generally behavioural problems rather than anxiety. Compulsions occur in these patients, mostly in presence of cerebral dysfunction and in the absence of obvious “ego-dystonic” qualities [9], as our patient does.

Our patient presented with prominent anxiety, obsessive doubts, and verification behaviour trying to confirm if he had done something wrong.

Some guidelines recommend to investigate blood ammonia and blood homocysteine in the presence of atypical symptoms, mental confusion, catatonia, fluctuation of symptoms, unusual (or paradoxical) response to treatment, progressive cognitive change or in an acute onset, and lack of treatment efficacy [10]. However, in this case, it was the personal patient history that led us to investigate his homocysteine levels. The improvement of the obsessive-compulsive symptoms with the treatment of homocystinuria led us to conclude that they were a manifestation of the disease. Although at first it was an unexpected finding, the diagnosis ended up being relatively easy.

Even though there are documented psychiatric symptoms induced by increased Hcy levels, the underlying mechanism is not well understood. Homocysteine is a sulfur-containing, nonprotein, toxic amino acid found in the pathway for the interconversion of two amino acids: methionine and cysteine. Homocysteine is metabolized via two different pathways: remethylation and transsulfuration. In the transsulfuration pathway, homocysteine is converted in cystathionine via vitamin B6-dependent enzyme cystathionine *β*-synthase (CBS), which requires pyridoxal 5′-phosphate as a cofactor. In the folate-dependent pathway, Hcy acquires a methyl group from N-5-methyltetrahydrofolate through methionine synthase and is converted in methionine. Methionine synthase requires vitamin B12 for its functionality, and the reaction also involves the recycling of tetrahydrofolate (from N-5-methyltetrahydrofolate) [11]. Homocysteine metabolism is intimately linked with the metabolism of folate, vitamin B12 (cobalamin), and vitamin B6 (pyridoxine). It is hypothesised that the pathogenesis of neuropsychiatric manifestations in homocystinuria, folate, and cobalamin deficiencies is related to imbalanced neurotransmitters in the central nervous system through disturbances in the pathways linking the metabolism of homocysteine and these vitamins [4]. Hcy is toxic to neuronal cells. By binding to the NMDA receptor, Hcy indirectly enhances calcium influx. Hcy has been found to induce neurological dysfunction via oxidative stress and can increase neuronal death and DNA damage. Hypomethylation of DNA and altered gene expression is two important mechanisms that lead to neuronal damage caused by elevated Hcy [12].

Present treatment strategies for homocystinuria include supplementation of vitamin B6/pyridoxine (100-200 mg/day), vitamin B12/cyanocobalamin (5 mg monthly), folic acid (5 mg/day), betaine (6-9 g/day), and intake of methionine-restricted diet [13]. Dietary treatment should be considered for all patients with CBS deficiency unless target Hcy levels are achieved entirely by pyridoxine supplementation. Diet may be used either as a sole treatment or adjunctive therapy along with pyridoxine [14].

The preconized treatment with pyridoxine (until 600 mg/day) and a low protein diet was our choice to treat our patient. In fact, after two weeks of treatment, the symptoms were practically absent, and in a month, he was asymptomatic. As the patient was pyridoxine responsive, it can explain his presentation at age of 39 and the relatively mild phenotype, in accordance with Abbott findings [3].

Even though psychiatric symptoms are frequent in homocystinuria, OCD-like symptoms are not that common. However, as a treatable condition, and easily diagnosed, it should be considered more often by psychiatrists.

## 4. Conclusion

Our case highlights a rare manifestation of homocystinuria with obsessive-compulsive symptoms. Due to his low prevalence, however, homocystinuria may be sometimes disregarded by psychiatrists when investigating their patients. Some findings may be red flags to it like past or family history of homocystinuria, mental retardation, thromboembolic episodes, vascular diseases or clinical, and laboratorial features resembling folate and/or vitamin B12 deficiencies. Further research on this subject may contribute to a better understanding of the underlying mechanisms relating homocysteine pathways and homocystinuria to obsessive-compulsive symptoms.
